# AI-Driven Prediction of Symptom Trajectories in Cancer Care: A Deep Learning Approach for Chemotherapy Management

**DOI:** 10.3390/bioengineering11111172

**Published:** 2024-11-20

**Authors:** Joseph Finkelstein, Aref Smiley, Christina Echeverria, Kathi Mooney

**Affiliations:** 1 Department of Biomedical Informatics, The University of Utah, Salt Lake City, UT 84108, USA; joseph.finkelstein@utah.edu; 2 College of Nursing, The University of Utah, Salt Lake City, UT 84112, USA; christina.echeverria@nurs.utah.edu (C.E.); kathi.mooney@nurs.utah.edu (K.M.)

**Keywords:** symptom prediction, deep learning, chemotherapy, symptom management

## Abstract

This study presents an advanced method for predicting symptom escalation in chemotherapy patients using Long Short-Term Memory (LSTM) networks and Convolutional Neural Networks (CNNs). The accurate prediction of symptom escalation is critical in cancer care to enable timely interventions and improve symptom management to enhance patients’ quality of life during treatment. The analytical dataset consists of daily self-reported symptom logs from chemotherapy patients, including a wide range of symptoms, such as nausea, fatigue, and pain. The original dataset was highly imbalanced, with approximately 84% of the data containing no symptom escalation. The data were resampled into varying interval lengths to address this imbalance and improve the model’s ability to detect symptom escalation (n = 3 to n = 7 days). This allowed the model to predict significant changes in symptom severity across these intervals. The results indicate that shorter intervals (n = 3 days) yielded the highest overall performance, with the CNN model achieving an accuracy of 81%, precision of 87%, recall of 80%, and an F1 score of 83%. This was an improvement over the LSTM model, which had an accuracy of 79%, precision of 85%, recall of 79%, and an F1 score of 82%. The model’s accuracy and recall declined as the interval length increased, though precision remained relatively stable. The findings demonstrate that both CNN’s temporospatial feature extraction and LSTM’s temporal modeling effectively capture escalation patterns in symptom progression. By integrating these predictive models into digital health systems, healthcare providers can offer more personalized and proactive care, enabling earlier interventions that may reduce symptom burden and improve treatment adherence. Ultimately, this approach has the potential to significantly enhance the overall quality of life for chemotherapy patients by providing real-time insights into symptom trajectories and guiding clinical decision making.

## 1. Introduction

Managing symptoms in cancer care is paramount for improving patients’ quality of life while undergoing treatment. Symptom management strategies aim to reduce the severity of symptoms and minimize the side effects of cancer therapies, enhancing both patient comfort and treatment outcomes [1]. The specific symptoms experienced by cancer patients can vary widely, depending on the type and stage of the cancer, as well as the treatments administered. Common symptoms include pain, fatigue, nausea, vomiting, and appetite changes [2]. According to the National Cancer Institute, 20% to 50% of cancer patients experience pain during their illness or treatment, while fatigue affects between 14% and 96% of patients undergoing treatment [2]. Addressing these symptoms requires a multidisciplinary approach that leverages evidence-based strategies and the combined efforts of healthcare professionals [3].

Previous randomized controlled trials demonstrated the positive impact of symptom monitoring on the quality of life of patients undergoing cancer treatment [4,5,6]. Chemotherapy, one of the most frequently used cancer treatments, targets rapidly dividing cancer cells but often causes significant side effects, including fatigue, nausea, vomiting, mouth ulcers, hair loss, and an increased risk of infections [7]. These side effects can substantially impact the patient’s quality of life and their ability to complete treatment. Therefore, effective symptom management is crucial to mitigating these adverse effects, improving patient outcomes, and increasing the likelihood of treatment adherence [1]. Recent studies have shown that continuous monitoring of patient-reported symptoms can significantly extend patient survival by enabling timely interventions, early detection of cancer recurrence, and better management of chemotherapy-related side effects [8].

In recent years, integrating digital health technologies in clinical workflows has emerged as a promising solution for improving symptom management in cancer care [9]. Home-based telemonitoring was shown to be well accepted by patients with chronic health conditions [10,11] and older adults [12]. Modern digital health technologies enable real-time monitoring of symptoms, offer automated guidance to patients and caregivers, and alert healthcare professionals to worsening conditions, allowing for timely interventions [13,14]. The deployment of such digital systems has shown great potential in addressing the complex and evolving needs of cancer patients, their caregivers, and healthcare providers [15]. However, a significant challenge remains in effectively utilizing patient-reported outcomes (PROs) to predict symptom escalation and determine when intervention is necessary [16].

Current criteria for identifying when symptom management protocols should be triggered often need to be more specific, leading to uncertainty about when to act [17,18]. To address this issue, machine learning (ML) techniques, particularly deep learning models like Long Short-Term Memory (LSTM) networks [19,20,21], have shown considerable promise in predicting cancer symptom trajectories based on sequential self-reported data [22]. LSTMs are particularly well suited for modeling temporal dependencies in patient-reported data and, therefore, the detection of patterns that may indicate an impending worsening of symptoms [23,24,25]. In contrast, CNNs leverage spatial feature extraction, making them effective in identifying symptom escalation patterns within the dataset’s complex structure [26,27,28].

Previous studies demonstrated the potential of deep learning models, such as LSTM and CNN, for predictive modeling of patient-generated data [29,30,31]. This study investigates both LSTM and CNN models to predict symptom escalation or prognosis in chemotherapy patients using longitudinal data from self-reported symptom logs. By leveraging sequential data over varying time intervals, each model captures distinct aspects of symptom progression: LSTMs excel in temporal pattern recognition, while CNNs enhance feature detection and analyses of data with grid-like topology. By enhancing the ability to predict symptom escalation in cancer patients, we can enable timely interventions and improve symptom management. Integrating these predictive models into digital health systems allows healthcare providers to deliver more personalized and proactive care, potentially improving patient outcomes and quality of life during cancer treatment.

## 2. Materials and Methods

### 2.1. Data Collection and Preprocessing

The analysis was conducted using data from the comprehensive Symptom Care at Home Study [32], which meticulously collected 26,599 records during chemotherapy treatment for cancer patients. Participants were asked to maintain a daily log of their symptoms through a technology-based system, ensuring a high level of compliance. For this study, 12 specific symptoms were collected, including nausea/vomiting, sore mouth, diarrhea, fatigue, trouble thinking, feeling blue, nervous/anxious, hair loss/weight change, pain/discomfort, numbness/tingling, trouble sleeping, and fever-related distress.

For each symptom, two metrics were recorded: severity and distress. If a symptom was not present, both values were marked as zero. For symptoms that were reported, each metric was scored from 1 to 10, where 10 represented the highest intensity. The only exception was fever, where only distress was measured. The overall symptom score was calculated by summing all symptoms’ severity and distress values, yielding a total possible score ranging from 0 to 230. This allowed for a quantifiable measure of the patient’s overall symptom burden. A total of 349 patients participated in this study. The median number of chemotherapy cycles per patient was three, with a range from one to thirteen cycles. On average, patients participated in the symptom survey for 78.24 days, with a median of 81 days and a maximum of 175 days. Data from patients who participated for fewer than three days, affecting ten patients, were excluded from the analysis. The final dataset included 339 patients.

Some missing values were observed for the severity and distress metrics across various symptoms. Linear interpolation was used to address these gaps, estimating the missing values using data from the same symptom recorded on the preceding and subsequent days. Finally, a combined ‘Severity + Distress’ score (for each symptom) was calculated for each day by summing the scores of all reported symptoms. This daily total score was then added to the dataset. The final dataset was highly imbalanced, with approximately 84% of the total scores being zero.

To address this imbalance, one approach was to define intervals within the data. By aggregating data into larger time intervals, we aimed to reduce the frequency of zero scores and provide the model with a more balanced distribution of the target variable. This method allowed us to capture meaningful trends across multiple days and improve the model’s ability to detect changes in symptom patterns.

### 2.2. Resampling and Interval Creation

The core of this method involves resampling the symptom data into n-day intervals. For each patient, data were grouped into intervals of 3, 4, 5, 6, or 7 days, and the average values of the features (i.e., symptom scores) were computed within each interval. This transformation condenses the daily data into manageable chunks while preserving temporal trends across intervals.

To ensure sufficient temporal data for each participant, only those with a minimum time span of n days × 7 intervals were included in the analysis, where n represents the number of days for interval resampling. Participants with shorter spans were excluded from the study to maintain consistency in interval-based resampling. In other words, patients were excluded if they had fewer than 21 days of continuous data for n = 3, and so on. The dataset was, thus, filtered to retain patients with sufficiently long time-series data, ensuring that all participants had at least n data intervals available for analysis. For example, if n = 3, participants must have at least 21 days of continuous data. We selected interval lengths of n = 3 to n = 7 days based on the goal of capturing both short- and medium-term symptom trends. Intervals shorter than 3 days, such as 2-day intervals, were deemed too brief to capture meaningful changes in symptom escalation. Longer intervals, such as 10 days, risked losing temporal resolution and responsiveness to changes, as symptoms could escalate rapidly within this period without timely detection

To address missing data points (such as when patients skipped reporting symptoms on some days), gaps in the resampled intervals were filled by averaging the surrounding valid intervals. This approach ensures that no significant gaps disrupt the temporal sequence, which is crucial for training the LSTM model.

**Figure 1:**
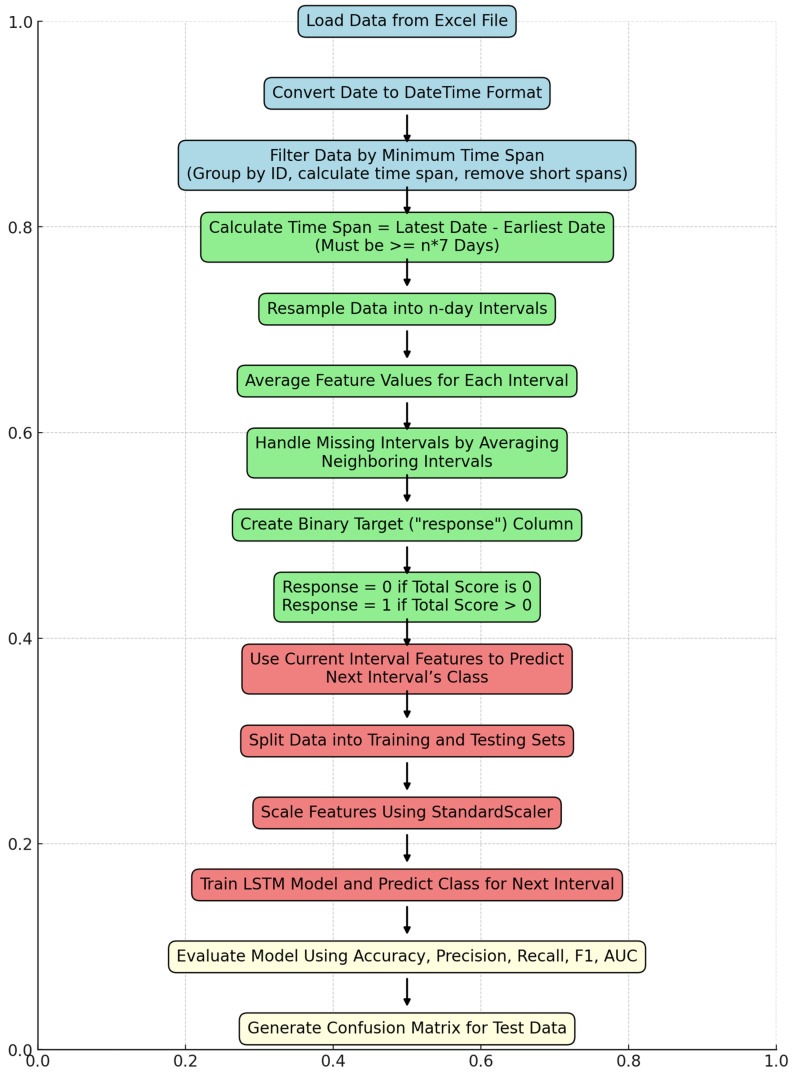
Flowchart of the developed algorithm to evaluate data. Final evaluation of compiled data.

A binary response column was created for each interval, where the response was coded as 1 if the total symptom score for that interval was greater than 0, and 0 otherwise. This binary classification framework was used to predict whether a patient would experience symptom escalation in the next interval. Each interval was assigned the response value of the subsequent interval to transform it into a prediction algorithm (e.g., using data from intervals 1 to 3, the model predicted whether symptoms would escalate in interval 4). Figure 1 provides an overview of the steps involved in training and testing the data.

### 2.3. Model Architecture: LSTM and CNN Approaches

Two deep learning models were applied to predict symptom escalation: an LSTM model to capture temporal dependencies and a CNN model to identify spatial patterns within the symptom data.

**LSTM Network:** An LSTM network was employed to model the temporal dependencies in the symptom data. The architecture of the LSTM was designed to capture both short-term fluctuations and longer-term trends in symptom scores, which are crucial for accurate forecasting.

- Input Layer: The data were reshaped into a 3D structure suitable for LSTM input (samples, timesteps, features), a common requirement for LSTM networks to capture temporal patterns. Each sample corresponds to a single patient record, with symptom data segmented into intervals of n days to represent sequential information. Each interval acts as a timestep, capturing the progression of symptom scores over time. The features dimension represents the various symptom metrics recorded for each interval, including severity and distress scores for each symptom. The model can learn temporal dependencies across intervals by reshaping the data into this 3D structure.
- LSTM Layer: The LSTM layer is the core of the model, with 50 units used to capture the temporal patterns within the data.
- Dropout Layer: To prevent overfitting, a dropout layer was added with a dropout rate of 0.2.
- Dense Layer: A fully connected layer with a sigmoid activation function was used to produce the binary classification output (i.e., whether symptoms would escalate or not in the next interval).

The model was compiled using binary cross-entropy as the loss function, with accuracy as the primary evaluation metric. The model was trained for 50 epochs with an 80–20 split for training and testing data. The Adam optimizer was used to ensure efficient and adaptive learning during model training.

**CNN Model:** The CNN model was structured to detect spatial patterns in symptom escalation by processing the data as sequences of symptom profiles.

- Convolutional Layer: A 1D convolutional layer with 64 filters and a kernel size of 3 was applied to capture local patterns within the symptom data sequence.
- Max-Pooling Layer: A max-pooling layer with a pool size of 2 was added to reduce spatial dimensions and retain prominent features.
- Flatten Layer: The features were then flattened into a vector for processing by fully connected layers.
- Dense Layer: A fully connected dense layer with 64 units and ReLU activation was added, followed by a dropout layer to guard against overfitting.
- Output Layer: The final output layer with a sigmoid activation function performed binary classification to predict symptom escalation.

The CNN model was compiled with binary cross-entropy as the loss function and used the Adam optimizer. Training was conducted with an 80–20 data split.

The performance of both models was evaluated using several metrics, including accuracy, precision, recall, F1 score, and the area under the receiver operating characteristic curve (AUC). These metrics provide a comprehensive view of the models’ ability to correctly predict symptom escalation, particularly in the context of class imbalance between symptom escalation (1 s) and non-escalation (0 s). Hyperparameter tuning was conducted using a grid search, adjusting parameters such as the number of units, learning rate, and dropout rate to achieve optimal performance.

This study was approved by the University of Utah Internal Review Board (protocol IRB_00017472).

## 3. Results

The performance of both the LSTM and CNN models was evaluated across different interval lengths (n = 3 to n = 7 days). These models allowed for comparisons in how each approach captured patterns in symptom escalation under varying temporal aggregations. Table 1 summarizes the performance metrics for each value of n, comparing results across the two models.

**Figure 2:**
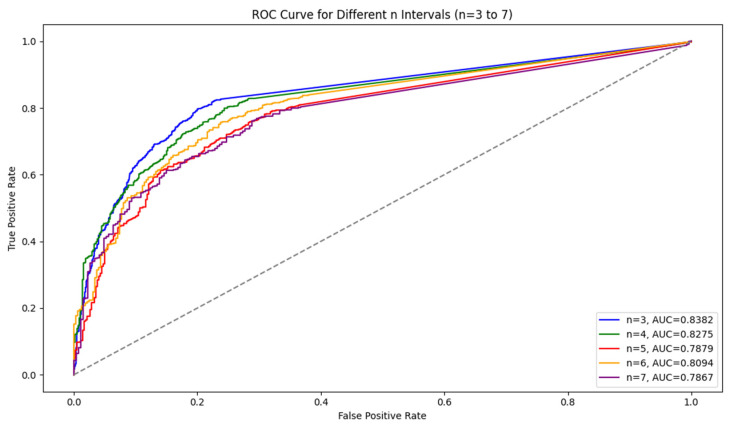
Comparison of the ROC curves for different n (3–7).

For the LSTM model, the results show that as the interval length increases, the model’s accuracy and recall decline, but precision remains stable. Shorter intervals (n = 3) capture symptom escalation patterns more effectively, providing the highest overall performance. In addition, Figure 2 shows a comparison of the ROC curve. We observed that AUC values tended to decrease as the interval length increased, indicating a decline in the model’s ability to distinguish between symptom escalation and non-escalation over longer time frames. This decline suggests that the model’s generalization ability weakens as n grows. Shorter intervals, with higher AUC values, allow the model to capture sharper, more immediate changes in symptom severity, resulting in better classification performance.

In contrast, the CNN model also showed superior performance at shorter intervals, achieving an accuracy of 81%, precision of 87%, recall of 80%, and an F1 score of 83% for n = 3. While both models demonstrated effective pattern recognition at shorter intervals, the CNN model consistently outperformed the LSTM model by a slight margin in accuracy, precision, and recall across most intervals.

The observed decline in model performance, particularly in accuracy and recall, as interval length n increases may be influenced by several factors. One primary reason is the reduction in available training data for longer intervals, which limits the model’s exposure to diverse patterns of symptom escalation. Additionally, longer intervals may dilute symptom escalation trends, as they average symptom scores over extended periods, potentially masking short-term fluctuations indicative of escalation. In contrast, shorter intervals preserve these temporal dynamics, allowing the model to detect abrupt changes more effectively. Another factor could be the nature of symptom escalation itself, which may not follow a steady progression and could vary widely between patients. Thus, larger intervals might fail to capture subtle but clinically relevant symptom changes that are more apparent in shorter time frames.

As n increased, the number of training and testing data points decreased, and more participant IDs were removed due to insufficient time spans. For instance, when n = 3, there were 8178 training data points and 40 IDs removed. At n = 7, the number of training data points dropped to 3384, and 74 IDs were removed. This is due to the need for longer time spans to generate larger intervals, which reduces the number of eligible participants for training.

Table 2 shows the total number of training data, testing data, and removed IDs for each value of n (the length of the intervals).

## 4. Discussion

The results of this study underscore the potential of LSTM and CNN in accurately predicting symptom escalation in chemotherapy patients. The models’ performance was most robust when using shorter time intervals (n = 3 days). For the LSTM model, shorter intervals proved especially effective in identifying temporal dependencies, as indicated by higher accuracy and recall. Similarly, the CNN model performed well with shorter intervals by leveraging spatial features within the data structure and showed slight improvements in precision and recall over LSTM. However, as the interval length increased, the model accuracy and recall exhibited a noticeable decline, while precision remained relatively stable. This suggests that shorter intervals are more suitable for predicting rapid changes in symptoms, a common occurrence during chemotherapy treatment.

These findings suggest that the LSTM and CNN models can offer valuable insights into symptom progression, with each model contributing unique strengths. While LSTMs are effective at modeling sequential data, CNNs add the capability to detect grid-like features, which may enhance predictive accuracy in complex datasets. This dual approach could be adapted across various clinical applications, particularly when continuous patient monitoring is essential. Implementing these models in digital health systems could provide automated alerts to healthcare providers, enabling proactive symptom management and allowing providers to focus more on patients with a high risk for symptom escalation. This aligns with the growing trend of using digital health tools to enhance symptom management in cancer care, where early intervention has been shown to improve both clinical outcomes and patient satisfaction. One challenge faced in this study was the highly imbalanced nature of the dataset, with approximately 84% of the total symptom scores being zero. The method of resampling data into n-day intervals helped to mitigate the imbalance, but the decline in performance for longer intervals underscores the difficulty of predicting symptom escalation over extended periods. Future research should explore additional techniques to address data imbalance, such as oversampling or synthetic data generation, which could further improve the model’s ability to predict symptom escalation over varying time intervals.

Table 3 compares results for both LSTM and CNN models across different time windows in predicting symptom escalation. In this analysis, different time windows (n = 3, 4, and 5 days) represent the number of preceding days’ symptom data used to predict symptom escalation on the following day. By using symptom data from a fixed number of previous days, the models aim to detect patterns and trends that could indicate an impending escalation. For instance, with n = 3, the model considers symptom scores from days i, i − 1, and i − 2 to predict whether escalation will occur on day i + 1. Expanding to n = 4 or n = 5 includes additional days of history, potentially providing the model with more context to identify escalation trends.

An interesting trade-off emerges with these results: adjusting the class weighting values in the models could potentially enhance the detection of symptom escalation (Class I Predictive Value) but would likely result in a reduction in overall accuracy. By increasing the weight given to positive cases, the models can be encouraged to focus more on detecting symptom escalations, which is often desirable in clinical or symptom monitoring settings. However, this comes at a cost, as the models may start to misclassify more non-escalation cases as escalations, leading to a decline in overall accuracy. This trade-off highlights the balance that must be struck between maximizing sensitivity to symptom escalation and maintaining an acceptable level of general accuracy across all cases. Adjusting class weights could, thus, be strategically applied based on the specific requirements of the application, favoring sensitivity when escalation detection is a priority.

The relatively low accuracies in Table 3, even with the defined weighting values, can be attributed to the imbalanced nature of the dataset (16.7% escalation days vs. 83.3% non-escalation days). This imbalance makes it challenging for the models to accurately detect escalation instances, as they are heavily outnumbered by non-escalation instances. To address this, we used a resampling strategy that helped to significantly improve both overall accuracy and escalation detection by balancing the dataset.

Our results are well aligned with previous reports demonstrating the potential of deep learning for the predictive modeling of serial patient-generated data [28,29,30,31,33]. Despite these promising results, several limitations should be noted. This study relied on self-reported symptom data, which may be subject to biases such as underreporting or inconsistencies in daily logging. Future work could explore integrating objective physiological data [34,35], such as heart rate or activity levels, and data from electronic health records [36,37] to supplement self-reports and enhance prediction accuracy. Additionally, this study focused on a specific subset of chemotherapy patients, and the generalizability of the results to other patient populations or cancer treatment modalities requires further investigation.

## 5. Conclusions

This study demonstrates the feasibility and effectiveness of using the LSTM and CNN models to predict symptom escalation in chemotherapy patients. By applying deep learning to patient-reported symptom data, healthcare providers can deliver more personalized, proactive care, potentially improving patient outcomes and quality of life. Further research should explore optimizing the models for longer time intervals and addressing data imbalance.

Integrating these predictive models into clinical workflows holds great potential for enhancing patient care. Real-time symptom monitoring can enable healthcare teams to intervene early, reducing symptom severity and helping patients adhere to their treatment regimen. Further research should explore refining these models for even longer time intervals, addressing data imbalance, and adapting the models for broader clinical applications, thereby maximizing their impact in healthcare and patient management.

## Figures and Tables

**Table 1:** Results for both the LSTM and CNN models are presented across various values of n to showcase the performance metrics for each interval length.

n	Model	Accuracy	Precision	Recall	F1 Score	AUC
3	LSTM	0.79	0.85	0.79	0.82	0.84
3	CNN	0.81	0.87	0.8	0.83	0.85
4	LSTM	0.78	0.85	0.79	0.82	0.83
4	CNN	0.8	0.86	0.81	0.83	0.84
5	LSTM	0.75	0.83	0.78	0.8	0.79
5	CNN	0.77	0.85	0.79	0.81	0.81
6	LSTM	0.77	0.84	0.82	0.83	0.81
6	CNN	0.78	0.85	0.82	0.83	0.82
7	LSTM	0.75	0.84	0.78	0.81	0.79
7	CNN	0.76	0.85	0.79	0.81	0.8

**Table 2:** Total number of data after applying n interval to each patient ID.

n	# of Training Data	# of Testing Data (20%)	Removed IDs
**3**	8178	2045	40
**4**	6142	1536	45
**5**	4918	1230	51
**6**	4032	1008	63
**7**	3384	846	74

**Table 3:** Performance comparison of LSTM and CNN models across different time windows for predicting symptom escalation on the unbalanced dataset (without resampling).

Days (n)	Model	Accuracy (%)	Class I Predictive Value (%)
3	LSTM	59.6	61.3
4	LSTM	58	65.3
5	LSTM	57.7	68.3
3	CNN	59.1	60.5
4	CNN	56.3	66.6
5	CNN	58.6	61.8

## Data Availability

Datasets are available upon request.
